# Supplementary material for: Polygenic risk of obesity and BMI trajectories over 36 years: A longitudinal study of adult Finnish twins
Source: Obesity (Silver Spring). 2023 Nov 21;31(12):3086–94. doi: 10.1002/oby.23906 (PMC10947257; doi:10.1002/oby.23906)
Supplement: Supplementary file 1 — Data S1. Supporting Information. [file OBY-31-3086-s001.docx]

# Online Supporting Information

Table S1: Prevalence of BMI categories in Finland in 1975, 1981, 1990, and 2011 according to NCD-RisC statistics, age-standardized among adults aged 20+ (1)

|  | **BMI category prevalence in Finland** | | | |
| --- | --- | --- | --- | --- |
|  | 1975 | 1981 | 1990 | 2011 |
| Underweight, % | 2 | 2 | 1 | 1 |
| Healthy weight, % | 62 | 58 | 51 | 41 |
| Overweight, % | 29 | 32 | 35 | 37 |
| Obesity, % | 7 | 9 | 13 | 21 |
| Mean BMI, kg/m^2^ | 24.5 | 24.8 | 25.3 | 26.1 |

Values are percentages or mean. BMI = body mass index. BMI categories (kg/m^2^): underweight (<18.5), normal weight (18.5 to <25), overweight (25 to <30), obesity (30+).

Reference:

1. NCD-RisC. Worldwide trends in body-mass index, underweight, overweight, and obesity from 1975 to 2016: a pooled analysis of 2416 population-based measurement studies in 128.9 million children, adolescents, and adults. N. C. D. Risk Factor Collaboration. Lancet. 2017;390(10113):2627-42.

Table S2a: Unadjusted values of characteristics of twin individuals in bottom and top PRS deciles

| **Comparison of characteristics between twins in top and bottom PRS decile** | | | | | | | | | | | | | | | | |
| --- | --- | --- | --- | --- | --- | --- | --- | --- | --- | --- | --- | --- | --- | --- | --- | --- |
|  | n (female %) | |  | | Observed BMI (kg/m^2^) | |  | | ΔBMI (kg/m^2^) partial interaction | | | |  | |  | |
|  | Bottom PRS  decile | Top  PRS  decile |  |  | Bottom PRS decile | Top  PRS decile | Contrast | p-value |  |  | Contrast | p-value |  |  | Bottom PRS decile | Top  PRS decile |
| 1975 | 646 (55) | 645 (57) |  | 1975 | 22.1 (0.1) | 24.6 (0.1) | 2.5 (0.2) | <0.001 |  | 1975 vs 1981 | 0.3 (0.3) | 0.26 |  | PRS, z-score | -1.7 (0.4) | 1.8 (0.4) |
| 1981 | 612 (56) | 602 (58) |  | 1981 | 22.7 (0.1) | 25.6 (0.2) | 2.9 (0.2) | <0.001 |  | 1981 vs 1990 | 0.4 (0.3) | 0.18 |  | Predicted BMI 1975, kg/m^2^ | 21.8 (1.6) | 24.5 (1.6) |
| 1990 | 496 (56) | 473 (58) |  | 1990 | 23.7 (0.2) | 27.0 (0.2) | 3.3 (0.2) | <0.001 |  | 1990 vs 2011 | 1.4 (0.4) | 0.0012 |  |  |  |  |
| 2011 | 229 (57) | 237 (56) |  | 2011 | 24.8 (0.2) | 29.4 (0.2) | 4.7 (0.3) | <0.001 |  |  |  |  |  |  |  |  |

Values are frequency (percentages) or mean (Delta-method standard error [SE]) and p-values were derived from contrast tests and post-hoc tests of partial interaction from the repeated measures mixed-effects linear regressions. PRS = polygenic risk score, n = number of individuals, BMI = body mass index, ΔBMI = BMI difference.

Table S2b: Adjusted values of characteristics of twin individuals in bottom and top PRS deciles

| **Comparison of characteristics between twins in top and bottom PRS decile** | | | | | | | |
| --- | --- | --- | --- | --- | --- | --- | --- |
|  | Observed BMI (kg/m^2^) | |  | | ΔBMI (kg/m^2^) partial interaction | | |
|  | Bottom PRS decile | Top PRS decile | Contrast | p-value |  | Contrast | p-value |
| 1975 | 22.1 (0.1) | 24.6 (0.1) | 2.5 (0.2) | <0.001 | 1975 vs 1981 | 0.4 (0.1) | <0.001 |
| 1981 | 22.7 (0.2) | 25.6 (0.1) | 2.9 (0.2) | <0.001 | 1981 vs 1990 | 0.5 (0.2) | 0.0035 |
| 1990 | 23.8 (0.2) | 27.2 (0.2) | 3.4 (0.3) | <0.001 | 1990 vs 2011 | 0.9 (0.3) | 0.0017 |
| 2011 | 25.7 (0.3) | 29.9 (0.3) | 4.3 (0.4) | <0.001 |  |  |  |

Values are mean (Delta-method std. err.) and p-values were derived from contrast tests and post-hoc tests of partial interaction from the repeated measures mixed-effects linear regressions. PRS = polygenic risk score, n = number of individuals, BMI = body mass index, ΔBMI = BMI difference.

Table S3a: Unadjusted means of observed Body mass index values by polygenic risk score decile over time

|  |  | **Body mass index (kg/m^2^)** | | |  |
| --- | --- | --- | --- | --- | --- |
| PRS percentile | 1975 | 1981 | 1990 | 2011 | ΔBMI 1975–2011, kg/m^2^ |
| 0-10 | 22.1 (0.1) | 22.7 (0.1) | 23.7 (0.2) | 24.8 (0.2) | 2.7 [2.5, 2.9] |
| 10-20 | 22.3 (0.1) | 23.1 (0.1) | 24.2 (0.2) | 24.8 (0.2) | 2.4 [2.2, 2.6] |
| 20-30 | 22.6 (0.1) | 23.2 (0.1) | 24.1 (0.2) | 25.4 (0.2) | 2.8 [2.6, 3.0] |
| 30-40 | 22.9 (0.1) | 23.6 (0.1) | 24.5 (0.2) | 26.1 (0.2) | 3.2 [3.0, 3.4] |
| 40-50 | 23.2 (0.1) | 23.9 (0.1) | 24.8 (0.2) | 25.9 (0.2) | 2.7 [2.5, 2.9] |
| 50-60 | 23.2 (0.1) | 23.9 (0.1) | 24.9 (0.2) | 26.5 (0.2) | 3.3 [3.1, 3.5] |
| 60-70 | 23.2 (0.1) | 23.9 (0.1) | 25.0 (0.2) | 26.4 (0.2) | 3.2 [3.0, 3.4] |
| 70-80 | 23.7 (0.1) | 24.4 (0.1) | 25.6 (0.2) | 27.4 (0.2) | 3.7 [3.5, 3.9] |
| 80-90 | 24.2 (0.1) | 25.0 (0.1) | 26.3 (0.2) | 28.2 (0.2) | 4.1 [3.9, 4.3] |
| 90-100 | 24.6 (0.1) | 25.6 (0.1) | 27.0 (0.2) | 29.4 (0.2) | 4.8 [4.7, 5.0] |

BMI values are mean (Delta-method std. err.) and ΔBMI values are mean [95% confidence interval].

Table S3b: Adjusted means body mass index values by polygenic risk score decile over time.

|  | **Body mass index (kg/m^2^)** | | | |  |
| --- | --- | --- | --- | --- | --- |
| PRS percentile | 1975 | 1981 | 1990 | 2011 | ΔBMI 1975–2011, kg/m^2^ |
| 0-10 | 22.1 (0.1) | 22.7 (0.1) | 23.8 (0.2) | 25.7 (0.2) | 3.6 [3.4, 3.8] |
| 10-20 | 22.4 (0.1) | 23.2 (0.1) | 24.4 (0.2) | 26.0 (0.2) | 3.6 [3.4, 3.9] |
| 20-30 | 22.7 (0.1) | 23.3 (0.1) | 24.4 (0.2) | 26.6 (0.2) | 3.9 [3.7, 4.2] |
| 30-40 | 22.9 (0.1) | 23.7 (0.1) | 24.8 (0.2) | 27.2 (0.2) | 4.3 [4.1, 4.5] |
| 40-50 | 23.1 (0.1) | 23.8 (0.1) | 25.0 (0.2) | 27.1 (0.2) | 4.0 [3.7, 4.2] |
| 50-60 | 23.1 (0.1) | 23.9 (0.1) | 25.1 (0.2) | 27.6 (0.2) | 4.4 [4.2, 4.7] |
| 60-70 | 23.3 (0.1) | 24.0 (0.1) | 25.3 (0.2) | 27.5 (0.2) | 4.2 [3.9, 4.4] |
| 70-80 | 23.6 (0.1) | 24.5 (0.1) | 25.8 (0.2) | 28.4 (0.2) | 4.8 [4.5, 5.0] |
| 80-90 | 24.2 (0.1) | 25.0 (0.1) | 26.4 (0.2) | 29.2 (0.2) | 5.0 [4.8, 5.3] |
| 90-100 | 24.6 (0.1) | 25.6 (0.1) | 27.1 (0.2) | 30.0 (0.2) | 5.4 [5.2, 5.6] |

BMI values are mean (Delta-method std. err.) and ΔBMI values are mean [95% confidence interval].

Table S3c: Number of individuals by polygenic risk score decile over time.

| PRS percentile | 1975 | 1981 | 1990 | 2011 |
| --- | --- | --- | --- | --- |
| 0-10 | 646 | 612 | 496 | 229 |
| 10-20 | 646 | 604 | 479 | 211 |
| 20-30 | 645 | 616 | 484 | 206 |
| 30-40 | 646 | 612 | 489 | 218 |
| 40-50 | 644 | 605 | 464 | 196 |
| 50-60 | 647 | 616 | 462 | 197 |
| 60-70 | 645 | 606 | 473 | 236 |
| 70-80 | 645 | 608 | 487 | 212 |
| 80-90 | 645 | 596 | 483 | 212 |
| 90-100 | 645 | 602 | 473 | 237 |

Values are frequency.

Table S4a: Regression analyses between PRS_BMI_ and BMI in 8,309 MZ and DZ twin individuals

|  | β BMI ∼ PRS_BMI_ | R^2^ | P-value | PRS min | PRS max | PRS range | BMI over PRS range |
| --- | --- | --- | --- | --- | --- | --- | --- |
| PRS_BMI_ (z-score [95% CI]) | 0.71 [0.64; 0.78] | 0.049 | <0.001 | -3.48 | 3.68 | 7.16 | 5.1 [4.6; 5.7] |
| Intercept | 23.3 [23.3; 23.4] |  |  |  |  |  |  |

Beta (β) and [95% confidence interval, 95% CI], BMI = body mass index, PRS_BMI_ = polygenic risk score for body mass index, min = minimum, max = maximum

Table S4b: Regression analyses between PRS_BMI_ and BMI of 8,309 MZ and DZ twin individuals, corrected for age and sex

|  | β BMI | R^2^ | P-value | Min | Max | Range | BMI over range |
| --- | --- | --- | --- | --- | --- | --- | --- |
| PRS_BMI_ (z-score, [95% CI]) | 0.78 [0.73; 0.84] | 0.293 | <0.001 | -3.48 | 3.68 | 7.16 | 5.6 [5.2; 6.0] |
| Age, years | 0.15 [0.14; 0.15] |  | <0.001 | 18 | 75 | 57 | 8.4 |
| Sex | -1.53 [-1.64; -1.41] |  | <0.001 | 1 (men) | 2 (women) | 1 | -1.5 |
| Intercept | 20.6 [20.4; 20.9] |  | <0.001 |  |  |  |  |

Beta (β) and [95% confidence interval, 95% CI], BMI = body mass index, PRS_BMI_ = polygenic risk score for body mass index, min = minimum, max = maximum


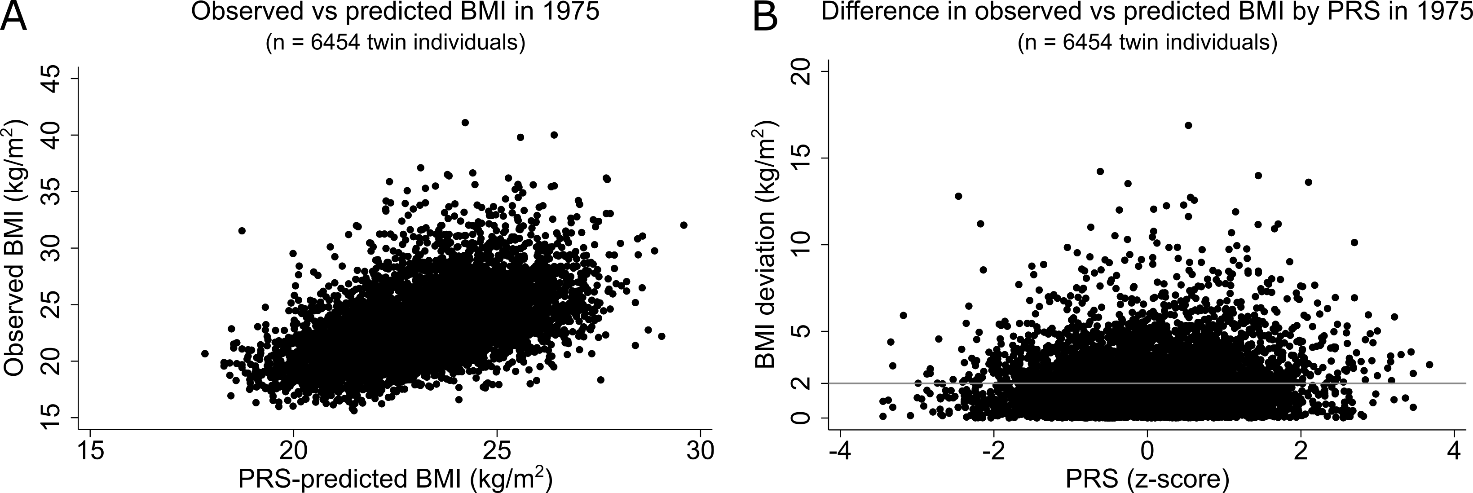
Figure S1: Scatterplots of observed body mass index (BMI) against polygenic risk score (PRS)-predicted BMI (A) and the absolute difference between observed and PRS-predicted BMI plotted against PRS (B) in all twin individuals from complete twin pairs in 1975. The gray line at 2.0 kg/m^2^ represents the average BMI deviation.


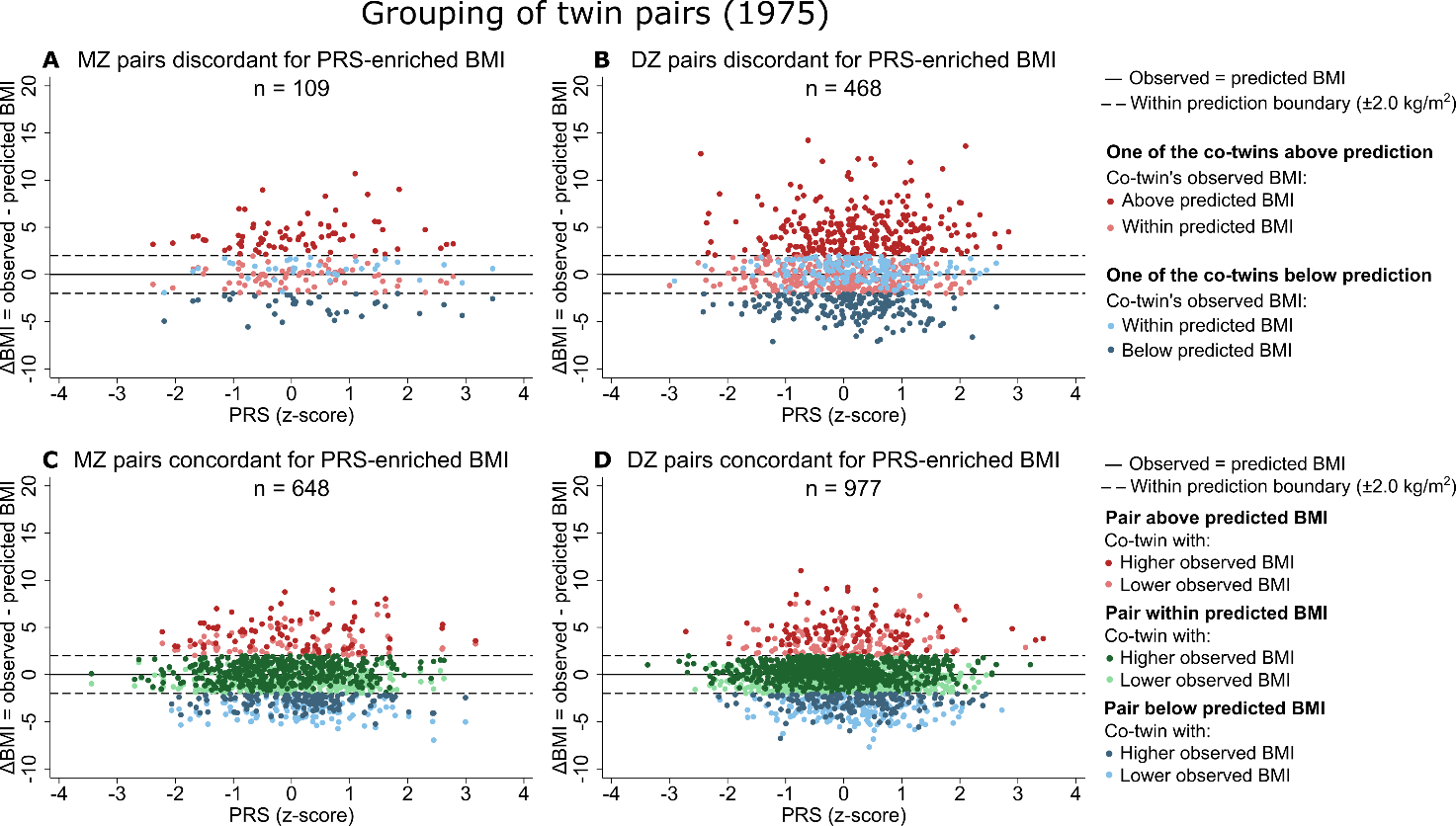


Figure S2: Categorizing the BMI difference (ΔBMI) between the observed body mass index (BMI) and the predicted BMI in monozygotic (MZ) and dizygotic (DZ) twin pairs discordant and concordant for PRS-enriched BMI (based on observed and predicted BMI values). n = number of pairs.

Table S5a: Number of twin pairs that participated per timepoint in subgroups of monozygotic and dizygotic twin pairs discordant for PRS-enriched BMI

|  | **Twin pairs discordant for PRS-enriched BMI** | | | |
| --- | --- | --- | --- | --- |
|  | MZ twin pairs  with one of the co-twin’s observed BMI | | DZ twin pairs  with one of the co-twin’s observed BMI | |
|  | Below prediction | Above prediction | Below prediction | Above prediction |
| 1975, n (female % of n) | 36 (64) | 73 (53) | 179 (57) | 289 (59) |
| 1981, n (female % of n) | 33 (64) | 69 (54) | 159 (53) | 249 (61) |
| 1990, n (female % of n) | 20 (60) | 49 (51) | 103 (56) | 182 (61) |
| 2011, n (female % of n) | 11 (64) | 23 (52) | 26 (69) | 57 (51) |

Values are frequency (percentage). PRS = polygenic risk score, BMI = body mass index, discordant for PRS-enriched BMI = large within-twin-pair differences in body mass index (≥ 3 kg/m^2^) considering both observed and predicted BMI, n = number of pairs, MZ = monozygotic, DZ = dizygotic.

Table S5b: Mean age of twins in subgroups of monozygotic and dizygotic twin pairs discordant for PRS-enriched BMI

|  | **Twin pairs discordant for PRS-enriched BMI** | | | |
| --- | --- | --- | --- | --- |
|  | MZ twin pairs  with one of the co-twin’s observed BMI | | DZ twin pairs  with one of the co-twin’s observed BMI | |
|  | Below prediction | Above prediction | Below prediction | Above prediction |
| 1975, years | 34 | 34 | 37 | 35 |
| 1981, years | 40 | 41 | 44 | 41 |
| 1990, years | 47 | 48 | 49 | 48 |
| 2011, years | 62 | 61 | 61 | 62 |

Values are mean. PRS = polygenic risk score, BMI = body mass index, discordant for PRS-enriched BMI = large within-twin-pair differences in body mass index (≥ 3 kg/m^2^) considering both observed and predicted BMI, MZ = monozygotic, DZ = dizygotic.

Table S6a: Number of twin pairs that participated per timepoint in subgroups of monozygotic and dizygotic twin pairs concordant for PRS-enriched BMI

|  | **Twin pairs concordant for PRS-enriched BMI** | | | | | |
| --- | --- | --- | --- | --- | --- | --- |
|  | MZ twin pairs’ observed BMI in both co-twins | | | DZ twin pairs’ observed BMI in both co-twins | | |
|  | Below  prediction | Within  prediction | Above  prediction | Below  prediction | Within  prediction | Above  prediction |
| 1975, n (female %) | 145 (66) | 463 (54) | 88 (63) | 162 (53) | 702 (52) | 113 (52) |
| 1981, n (female %) | 139 (68) | 433 (55) | 80 (64) | 149 (55) | 640 (54) | 105 (52) |
| 1990, n (female %) | 102 (69) | 337 (56) | 60 (62) | 100 (50) | 448 (54) | 71 (58) |
| 2011, n (female %) | 48 (63) | 210 (58) | 37 (73) | 32 (50) | 209 (54) | 24 (50) |

Values are frequency (percentage). BMI = body mass index, PRS = polygenic risk score, concordant for PRS-enriched BMI = small within-twin-pair differences in body mass index (< 3 kg/m^2^) considering both observed and predicted BMI, n = number of pairs, MZ = monozygotic, DZ = dizygotic.

Table S6b: Mean age of twins in subgroups of monozygotic and dizygotic twin pairs concordant for PRS-enriched BMI

|  | **Twin pairs concordant for PRS-enriched BMI** | | | | | |
| --- | --- | --- | --- | --- | --- | --- |
|  | MZ twin pairs’ observed BMI in both co-twins | | | DZ twin pairs’ observed BMI in both co-twins | | |
|  | Below  prediction | Within  prediction | Above  prediction | Below  prediction | Within  prediction | Above  prediction |
| 1975, years | 33 | 30 | 32 | 36 | 33 | 35 |
| 1981, years | 39 | 37 | 38 | 42 | 39 | 41 |
| 1990, years | 46 | 45 | 46 | 49 | 47 | 49 |
| 2011, years | 60 | 60 | 61 | 61 | 60 | 61 |

Values are mean. PRS = polygenic risk score, BMI = body mass index, concordant for PRS-enriched BMI = small within-twin-pair differences in body mass index (< 3 kg/m^2^) considering both observed and predicted BMI, MZ = monozygotic, DZ = dizygotic.

Table S7: Polygenic risk score and predicted BMI in 1975 in subgroups of MZ and DZ twin pairs discordant for PRS-enriched BMI

|  | **Twin pairs discordant for PRS-enriched BMI** | | | | | | | | |  |
| --- | --- | --- | --- | --- | --- | --- | --- | --- | --- | --- |
|  | MZ twin pairs with one of the co-twin’s observed BMI | | | DZ twin pairs with one of the co-twin’s observed BMI | | | | | | |
|  | Below prediction  (n = 36) | Above prediction  (n = 73) | Below prediction  (n = 179) | | |  | Above prediction  (n = 289) | |  | |
|  | Both co-twins below  and within prediction | Both co-twins above  and within prediction | Co-twin below  prediction | | Co-twin within  prediction | p-value | Co-twin within  prediction | Co-twin above  prediction | | p-value |
| PRS, z-score | 0.4 (1.3) | 0.1 (1.1) | 0.1 (0.9) | | 0.3 (0.9) | <0.001 | -0.1 (1.0) | 0.3 (1.0) | | <0.001 |
| Predicted BMI, kg/m^2^ | 23.5 (1.7) | 23.5 (1.6) | 23.8 (1.8) | | 24.0 (1.8) | 0.31 | 23.3 (1.6) | 23.6 (1.7) | | 0.022 |

Values are mean (SD) and t-tests gave p-values. BMI = body mass index, PRS = polygenic risk score, discordant for PRS-enriched BMI = large within-twin-pair differences in body mass index (≥ 3 kg/m^2^) considering both observed and predicted BMI, MZ = monozygotic, DZ = dizygotic, n = number of pairs, ΔBMI = observed BMI minus predicted BMI, |ΔBMI| = absolute values of observed BMI minus predicted BMI

Table S8: Polygenic risk scores and predicted BMI in 1975 in subgroups of MZ twin pairs concordant for PRS-enriched BMI

|  | **MZ twin pairs concordant for PRS-enriched BMI** | | |
| --- | --- | --- | --- |
|  | Both co-twins in MZ pairs  below prediction  (n = 136) | Both co-twins in MZ pairs within prediction  (n = 432) | Both co-twins in MZ pairs above prediction  (n = 80) |
| PRS, z-score | 0.1 (0.9) | -0.2 (1.0) | 0.0 (1.1) |
| Predicted BMI, kg/m^2^ | 23.1 (1.7) | 22.6 (1.7) | 22.9 (1.7) |

Values are mean (SD) and t-tests gave p-values. MZ = monozygotic, BMI = body mass index, PRS = polygenic risk score, concordant for PRS-enriched BMI = small within-twin-pair differences in body mass index (< 3 kg/m^2^) considering both observed and predicted BMI, n = number of pairs

Table S9: Polygenic risk scores and predicted BMI in 1975 in subgroups of DZ twin pairs concordant for PRS-enriched BMI

|  | **DZ twin pairs concordant for PRS-enriched BMI** | | | | | | | |  |
| --- | --- | --- | --- | --- | --- | --- | --- | --- | --- |
|  | DZ pair below prediction  (n = 162) | |  | DZ pair within prediction  (n = 702) | |  | DZ pair above prediction  (n = 113) | |  |
|  | Co-twin with  lower BMI | Co-twin with  higher BMI | p-value | Co-twin with  lower BMI | Co-twin with  higher BMI | p-value | Co-twin with  lower BMI | Co-twin with  higher BMI | p-value |
| PRS, z-score | 0.2 (0.9) | 0.2 (0.9) | 0.81 | -0.1 (1.0) | -0.1 (0.9) | 0.016 | 0.1 (1.0) | 0.1 (0.9) | 0.82 |
| Predicted BMI, kg/m^2^ | 23.7 (1.7) | 23.7 (1.7) | 0.96 | 23.0 (1.8) | 23.1 (1.7) | 0.59 | 23.6 (1.7) | 23.6 (1.7) | 0.96 |

Values are mean (SD) and t-tests gave p-values. DZ = dizygotic, BMI = body mass index, PRS = polygenic risk score, concordant for PRS-enriched BMI = small within-twin-pair differences in body mass index (< 3 kg/m^2^) considering both observed and predicted BMI, n = number of pairs.

Table S10a: Unadjusted values from contrast tests that compared observed body mass index at each timepoint within subgroups of monozygotic twin pairs discordant for PRS-enriched BMI

|  | **MZ twin pairs discordant for PRS-enriched BMI** | | | | | | | |
| --- | --- | --- | --- | --- | --- | --- | --- | --- |
|  | One of the MZ co-twin’s observed BMI | | | | | | | |
|  | Below prediction (n = 36) | |  | | Above prediction (n = 73) | |  | |
|  | Co-twin  below prediction | Co-twin  within prediction | Contrast | p-value | Co-twin  within prediction | Co-twin  above prediction | Contrast | p-value |
| 1975, kg/m^2^ | 20.2 (0.4) | 24.1 (0.4) | 3.8 (0.6) | <0.001 | 23.3 (0.4) | 27.8 (0.4) | 4.5 (0.5) | <0.001 |
| 1981, kg/m^2^ | 21.2 (0.4) | 24.0 (0.5) | 2.7 (0.6) | <0.001 | 24.7 (0.4) | 27.3 (0.4) | 2.5 (0.5) | <0.001 |
| 1990, kg/m^2^ | 21.3 (0.6) | 24.6 (0.5) | 3.3 (0.8) | <0.001 | 26.5 (0.4) | 28.4 (0.4) | 1.9 (0.6) | 0.002 |
| 2011, kg/m^2^ | 23.3 (0.8) | 26.4 (0.8) | 3.1 (1.1) | 0.006 | 28.2 (0.6) | 31.3 (0.6) | 3.1 (0.9) | 0.001 |
| ΔBMI 1975–2011, kg/m^2^ | 3.0 [2.4, 3.7] | 2.3 [1.6, 3.0] |  |  | 4.9 [4.4, 5.4] | 3.5 [3.0, 4.0] |  |  |
| ΔBMI 1975–2011, p-value | 0.001 | 0.011 |  |  | <0.001 | <0.001 |  |  |

BMI values are mean (Delta-method std. err.), ΔBMI values are mean [95% confidence interval], and contrast tests gave mean (SE) values and p-values. MZ = monozygotic, BMI = body mass index, PRS = polygenic risk score, discordant for PRS-enriched BMI = large within-twin-pair differences in body mass index (≥ 3 kg/m^2^) considering both observed and predicted BMI, n = number of pairs, ΔBMI = BMI_2011_ – BMI_1975_.

Table S10b: Adjusted values from contrast tests that compared observed body mass index at each timepoint within subgroups of monozygotic twin pairs discordant for PRS-enriched BMI

|  | **MZ twin pairs discordant for PRS-enriched BMI** | | | | | | | |
| --- | --- | --- | --- | --- | --- | --- | --- | --- |
|  | One of the MZ co-twin’s observed BMI | | | | | | | |
|  | Below prediction (n = 36) | |  |  | Above prediction (n = 73) | |  |  |
|  | Co-twin  below prediction | Co-twin  within prediction | Contrast | p-value | Co-twin  within prediction | Co-twin  above prediction | Contrast | p-value |
| 1975, kg/m^2^ | 20.2 (0.3) | 24.1 (0.3) | 3.8 (0.1) | <0.001 | 23.3 (0.2) | 27.8 (0.2) | 4.5 (0.2) | <0.001 |
| 1981, kg/m^2^ | 21.3 (0.5) | 24.0 (0.5) | 2.7 (0.5) | <0.001 | 24.8 (0.3) | 27.3 (0.3) | 2.5 (0.3) | <0.001 |
| 1990, kg/m^2^ | 21.9 (0.6) | 25.2 (0.5) | 3.3 (0.6) | <0.001 | 26.5 (0.5) | 28.6 (0.5) | 2.1 (0.6) | 0.001 |
| 2011, kg/m^2^ | 24.6 (1.0) | 27.8 (1.0) | 3.2 (1.1) | 0.003 | 28.7 (0.7) | 32.0 (0.7) | 3.2 (1.0) | 0.001 |
| ΔBMI 1975–2011, kg/m^2^ | 4.3 [3.1, 5.6] | 3.7 [2.5, 5.0] |  |  | 5.5 [4.5, 6.4] | 4.2 [3.2, 5.2] |  |  |
| ΔBMI 1975–2011, p-value | <0.001 | <0.001 |  |  | <0.001 | <0.001 |  |  |

BMI values are mean (Delta-method std. err.), ΔBMI values are mean [95% confidence interval], and contrast tests gave mean (SE) values and p-values. MZ = monozygotic, discordant for PRS-enriched BMI = large within-twin-pair differences in body mass index (≥ 3 kg/m^2^) considering both observed and predicted BMI, BMI = body mass index, PRS = polygenic risk score, n = number of pairs, ΔBMI = BMI_2011_ – BMI_1975_.

Table S11a: Unadjusted values from contrast tests that compared observed body mass index at each timepoint within subgroups of dizygotic twin pairs discordant for PRS-enriched BMI

|  | **DZ twin pairs discordant for PRS-enriched BMI** | | | | | | | |
| --- | --- | --- | --- | --- | --- | --- | --- | --- |
|  | One of the DZ co-twin’s observed BMI | | | | | | | |
|  | Below prediction (n = 179) | |  | | Above prediction (n = 289) | |  | |
|  | Co-twin  below prediction | Co-twin  within prediction | Contrast | p-value | Co-twin  within prediction | Co-twin  above prediction | Contrast | p-value |
| 1975, kg/m^2^ | 20.2 (0.2) | 24.4 (0.2) | 4.2 (0.3) | <0.001 | 23.0 (0.2) | 28.4 (0.2) | 5.3 (0.3) | <0.001 |
| 1981, kg/m^2^ | 21.2 (0.2) | 25.0 (0.2) | 3.8 (0.3) | <0.001 | 24.0 (0.2) | 28.9 (0.2) | 4.9 (0.3) | <0.001 |
| 1990, kg/m^2^ | 21.9 (0.2) | 26.0 (0.2) | 4.1 (0.3) | <0.001 | 25.0 (0.2) | 30.1 (0.2) | 5.2 (0.3) | <0.001 |
| 2011, kg/m^2^ | 23.8 (0.4) | 27.8 (0.4) | 4.0 (0.6) | <0.001 | 26.6 (0.4) | 32.0 (0.4) | 5.4 (0.5) | <0.001 |
| ΔBMI 1975–2011, kg/m^2^ | 3.5 [3.0, 4.0] | 3.4 [2.9, 3.9] |  |  | 3.6 [3.2, 3.9] | 3.6 [3.2, 4.0] |  |  |
| ΔBMI 1975–2011, p-value | <0.001 | <0.001 |  |  | <0.001 | <0.001 |  |  |

BMI values are mean (Delta-method std. err.), ΔBMI values are mean [95% confidence interval], and contrast tests gave mean (SE) values and p-values. DZ = dizygotic, discordant for PRS-enriched BMI = large within-twin-pair differences in body mass index (≥ 3 kg/m^2^) considering both observed and predicted BMI, BMI = body mass index, PRS = polygenic risk score, n = number of pairs, ΔBMI = BMI_2011_ – BMI_1975_.

Table S11b: Adjusted values from contrast tests that compared observed body mass index at each timepoint within subgroups of dizygotic twin pairs discordant for PRS-enriched BMI

|  | **DZ twin pairs discordant for PRS-enriched BMI** | | | | | | | |
| --- | --- | --- | --- | --- | --- | --- | --- | --- |
|  | One of the DZ co-twin’s observed BMI | | | | | | | |
|  | Below prediction (n = 179) | |  | | Above prediction (n = 289) | |  | |
|  | Co-twin  below prediction | Co-twin  within prediction | Contrast | p-value | Co-twin  within prediction | Co-twin  above prediction | Contrast | p-value |
| 1975, kg/m^2^ | 20.2 (0.1) | 24.4 (0.1) | 4.2 (0.1) | <0.001 | 23.0 (0.1) | 28.4 (0.1) | 5.3 (0.1) | <0.001 |
| 1981, kg/m^2^ | 21.2 (0.2) | 24.9 (0.2) | 3.7 (0.2) | <0.001 | 24.0 (0.2) | 28.8 (0.2) | 4.8 (0.2) | <0.001 |
| 1990, kg/m^2^ | 22.4 (0.3) | 26.3 (0.3) | 4.0 (0.4) | <0.001 | 25.3 (0.2) | 30.3 (0.2) | 5.0 (0.3) | <0.001 |
| 2011, kg/m^2^ | 25.7 (0.6) | 29.2 (0.6) | 3.5 (0.8) | <0.001 | 27.4 (0.4) | 32.6 (0.4) | 5.2 (0.6) | <0.001 |
| ΔBMI 1975–2011, kg/m^2^ | 5.5 [4.6, 6.3] | 4.8 [4.0, 5.6] |  |  | 4.3 [3.8, 4.9] | 4.2 [3.6, 4.8] |  |  |
| ΔBMI 1975–2011, p-value | <0.001 | <0.001 |  |  | <0.001 | <0.001 |  |  |

BMI values are mean (Delta-method std. err.), ΔBMI values are mean [95% confidence interval], and contrast tests gave mean (SE) values and p-values. DZ = dizygotic, discordant for PRS-enriched BMI = large within-twin-pair differences in body mass index (≥ 3 kg/m^2^) considering both observed and predicted BMI, BMI = body mass index, PRS = polygenic risk score, n = number of pairs, ΔBMI = BMI_2011_ – BMI_1975_.

Table S12a: Unadjusted values from post-hoc tests of partial interaction between co-twin subgroup and BMI between adjacent timepoints in monozygotic and dizygotic twin pairs discordant for PRS-enriched BMI

|  | **BMI differences between co-twins and adjacent timepoints in twin pairs discordant for PRS-enriched BMI** | | | | | | | |
| --- | --- | --- | --- | --- | --- | --- | --- | --- |
|  | MZ co-twins  below vs within  (n = 36) | | MZ co-twins  above vs within  (n = 73) | | DZ co-twins  below vs within  (n = 179) | | DZ co-twins  above vs within  (n = 289) | |
|  | Contrast | p-value | Contrast | p-value | Contrast | p-value | Contrast | p-value |
| 1975 vs 1981, kg/m^2^ | -1.1 (0.9) | 0.21 | -2.0 (0.8) | 0.009 | -0.3 (0.4) | 0.39 | -0.4 (0.4) | 0.25 |
| 1981 vs 1990, kg/m^2^ | 0.6 (1.0) | 0.56 | -0.6 (0.8) | 0.44 | 0.3 (0.4) | 0.45 | 0.3 (0.4) | 0.49 |
| 1990 vs 2011, kg/m^2^ | -0.2 (1.3) | 0.87 | 1.2 (1.1) | 0.27 | -0.1 (0.7) | 0.87 | 0.2 (0.6) | 0.76 |

Values are mean (SE) and post-hoc tests of partial interaction gave p-values. Discordant for PRS-enriched BMI = large within-twin-pair differences in body mass index (≥ 3 kg/m^2^) considering both observed and predicted BMI, BMI = body mass index, PRS = polygenic risk score, n = number of pairs, MZ = monozygotic, DZ = dizygotic.

Table S12b: Adjusted values from post-hoc tests of partial interaction between co-twin subgroup and BMI between adjacent timepoints in monozygotic and dizygotic twin pairs discordant for PRS-enriched BMI

|  | **BMI differences between co-twins and adjacent timepoints in twin pairs discordant for PRS-enriched BMI** | | | | | | | |
| --- | --- | --- | --- | --- | --- | --- | --- | --- |
|  | MZ co-twins  below vs within  (n = 36) | | MZ co-twins  above vs within  (n = 73) | | DZ co-twins  below vs within  (n = 179) | | DZ co-twins  above vs within  (n = 289) | |
|  | Contrast | p-value | Contrast | p-value | Contrast | p-value | Contrast | p-value |
| 1975 vs 1981, kg/m^2^ | -1.1 (0.5) | 0.015 | -2.0 (0.3) | <0.001 | -0.5 (0.2) | 0.004 | -0.5 (0.2) | 0.007 |
| 1981 vs 1990, kg/m^2^ | 0.6 (0.5) | 0.24 | -0.5 (0.5) | 0.37 | 0.3 (0.4) | 0.43 | 0.2 (0.2) | 0.48 |
| 1990 vs 2011, kg/m^2^ | -0.1 (1.2) | 0.95 | 1.2 (0.9) | 0.19 | -0.5 (0.8) | 0.55 | 0.2 (0.5) | 0.69 |

Values are mean (SE) and post-hoc tests of partial interaction gave p-values. Discordant for PRS-enriched BMI = large within-twin-pair differences in body mass index (≥ 3 kg/m^2^) considering both observed and predicted BMI, BMI = body mass index, PRS = polygenic risk score, n = number of pairs, MZ = monozygotic, DZ = dizygotic.

Table S13a: Unadjusted values from contrast tests that compared observed body mass index at each timepoint within subgroups of monozygotic twin pairs concordant for PRS-enriched BMI

| **Observed BMI (kg/m^2^) in MZ twin pairs concordant for PRS-enriched BMI** | | | | | | | | | | | | |
| --- | --- | --- | --- | --- | --- | --- | --- | --- | --- | --- | --- | --- |
|  | MZ pair below prediction  (n = 136) | | | | MZ pair within prediction  (n = 432) | | | | MZ pair above prediction  (n = 80) | | | |
|  | Co-twin with  lower BMI | Co-twin with  higher BMI | Contrast | p-value | Co-twin with  lower BMI | Co-twin with  higher BMI | Contrast | p-value | Co-twin with  lower BMI | Co-twin with  higher BMI | Contrast | p-value |
| 1975 | 19.4 (0.2) | 20.2 (0.2) | 0.9 (0.3) | 0.001 | 22.0 (0.1) | 22.9 (0.1) | 0.9 (0.2) | <0.001 | 26.3 (0.4) | 27.5 (0.4) | 1.2 (0.6) | 0.028 |
| 1981 | 20.6 (0.2) | 20.8 (0.2) | 0.2 (0.3) | 0.37 | 23.0 (0.1) | 23.7 (0.1) | 0.7 (0.2) | <0.001 | 27.2 (0.4) | 27.7 (0.4) | 0.5 (0.6) | 0.35 |
| 1990 | 21.5 (0.2) | 21.6 (0.2) | 0.04 (0.3) | 0.88 | 24.0 (0.1) | 24.8 (0.1) | 0.7 (0.2) | <0.001 | 28.6 (0.4) | 29.3 (0.4) | 0.7 (0.6) | 0.27 |
| 2011 | 23.3 (0.3) | 23.6 (0.3) | 0.2 (0.4) | 0.59 | 25.9 (0.2) | 26.2 (0.2) | 0.3 (0.3) | 0.18 | 29.0 (0.6) | 30.6 (0.6) | 1.6 (0.8) | 0.062 |
| ΔBMI | 4.0 [3.7, 4.2] | 3.3 [3.1, 3.6] |  |  | 3.9 [3.8, 4.0] | 3.3 [3.2, 3.4] |  |  | 2.7 [2.3, 3.1] | 3.1 [2.7, 3.5] |  |  |
| ΔBMI *P* | <0.001 | <0.001 |  |  | <0.001 | <0.001 |  |  | <0.001 | <0.001 |  |  |

BMI values are mean (Delta-method std. err.), ΔBMI values are mean [95% confidence interval], and contrast tests gave mean (SE) values and p-values. BMI = body mass index, MZ = monozygotic, concordant for PRS-enriched BMI = small within-twin-pair differences in body mass index (< 3 kg/m^2^) considering both observed and predicted BMI, PRS = polygenic risk score, n = number of pairs, ΔBMI = BMI_2011_ – BMI_1975_, ΔBMI P = p-values for BMI_2011_ – BMI_1975_ by co-twin group.

Table S13b: Adjusted values from contrast tests that compared observed body mass index at each timepoint within subgroups of monozygotic twin pairs concordant for PRS-enriched BMI

| **Observed BMI (kg/m^2^) in MZ twin pairs concordant for PRS-enriched BMI** | | | | | | | | | | | | |
| --- | --- | --- | --- | --- | --- | --- | --- | --- | --- | --- | --- | --- |
|  | MZ pair below prediction  (n = 136) | | | | MZ pair within prediction  (n = 432) | | | | MZ pair above prediction  (n = 80) | | | |
|  | Co-twin with  lower BMI | Co-twin with  higher BMI | Contrast | p-value | Co-twin with  lower BMI | Co-twin with  higher BMI | Contrast | p-value | Co-twin with  lower BMI | Co-twin with  higher BMI | Contrast | p-value |
| 1975 | 19.4 (0.1) | 20.2 (0.1) | 0.9 (0.1) | <0.001 | 22.0 (0.1) | 22.9 (0.1) | 0.9 (0.04) | <0.001 | 26.3 (0.3) | 27.5 (0.3) | 1.2 (0.1) | <0.001 |
| 1981 | 20.6 (0.2) | 20.8 (0.2) | 0.2 (0.1) | 0.19 | 23.0 (0.1) | 23.7 (0.1) | 0.7 (0.1) | <0.001 | 27.2 (0.3) | 27.7 (0.3) | 0.4 (0.3) | 0.22 |
| 1990 | 21.8 (0.2) | 21.8 (0.2) | -0.03 (0.2) | 0.88 | 24.1 (0.1) | 24.8 (0.1) | 0.7 (0.1) | <0.001 | 28.6 (0.4) | 29.2 (0.4) | 0.5 (0.5) | 0.30 |
| 2011 | 23.6 (0.4) | 24.4 (0.4) | 0.8 (0.6) | 0.13 | 26.5 (0.2) | 26.9 (0.2) | 0.5 (0.3) | 0.11 | 30.5 (0.7) | 31.8 (0.7) | 1.4 (0.9) | 0.11 |
| ΔBMI | 4.2 [3.7, 4.7] | 4.2 [3.6, 4.7] |  |  | 4.4 [4.2, 4.7] | 4.0 [3.7, 4.3] |  |  | 4.2 [3.4, 5.0] | 4.4 [3.6, 5.1] |  |  |
| ΔBMI *P* | <0.001 | <0.001 |  |  | <0.001 | <0.001 |  |  | <0.001 | <0.001 |  |  |

BMI values are mean (Delta-method std. err.), ΔBMI values are mean [95% confidence interval], and contrast tests gave mean (SE) values and p-values. BMI = body mass index, MZ = monozygotic, concordant for PRS-enriched BMI = small within-twin-pair differences in body mass index (< 3 kg/m^2^) considering both observed and predicted BMI, PRS = polygenic risk score, n = number of pairs, ΔBMI = BMI_2011_ – BMI_1975_, ΔBMI P = p-values for BMI_2011_ – BMI_1975_ by co-twin group.

Table S14a: Unadjusted values from contrast tests that compared observed body mass index at each timepoint within subgroups of dizygotic twin pairs concordant for PRS-enriched BMI

| **Observed BMI (kg/m^2^) in DZ twin pairs concordant for PRS-enriched BMI** | | | | | | | | | | | | | |
| --- | --- | --- | --- | --- | --- | --- | --- | --- | --- | --- | --- | --- | --- |
|  | DZ pair below prediction  (n = 162) | | | | DZ pair within prediction  (n = 702) | | | | DZ pair above prediction  (n = 113) | | |  |  |
|  | Co-twin with  lower BMI | Co-twin with  higher BMI | Contrast | p-value | Co-twin with  lower BMI | Co-twin with  higher BMI | Contrast | p-value | Co-twin with  lower BMI | Co-twin with  higher BMI | Contrast | p-value |  |
| 1975 | 19.9 (0.2) | 20.8 (0.2) | 0.9 (0.2) | <0.001 | 22.4 (0.1) | 23.4 (0.1) | 1.0 (0.1) | <0.001 | 27.0 (0.3) | 28.3 (0.3) | 1.2 (0.4) | 0.002 |  |
| 1981 | 20.6 (0.2) | 21.6 (0.2) | 0.9 (0.2) | <0.001 | 23.2 (0.1) | 24.0 (0.1) | 0.7 (0.1) | <0.001 | 27.9 (0.3) | 28.6 (0.3) | 0.7 (0.4) | 0.10 |  |
| 1990 | 21.8 (0.2) | 22.6 (0.2) | 0.7 (0.3) | 0.007 | 24.3 (0.1) | 25.0 (0.1) | 0.7 (0.2) | <0.001 | 28.8 (0.3) | 29.6 (0.3) | 0.8 (0.5) | 0.071 |  |
| 2011 | 23.8 (0.3) | 24.5 (0.3) | 0.7 (0.5) | 0.12 | 25.9 (0.2) | 26.5 (0.2) | 0.6 (0.2) | 0.009 | 30.7 (0.6) | 31.2 (0.6) | 0.5 (0.8) | 0.56 |  |
| ΔBMI | 3.9 [3.6, 4.2] | 3.7 [3.4, 4.0] |  |  | 3.5 [3.3, 3.6] | 3.1 [2.9, 3.2] |  |  | 3.7 [3.1, 4.2] | 2.9 [2.3, 3.4] |  |  |  |
| ΔBMI *P* | <0.001 | <0.001 |  |  | <0.001 | <0.001 |  |  | <0.001 | <0.001 |  |  |  |

BMI values are mean (Delta-method std. err.), ΔBMI values are mean [95% confidence interval], and contrast tests gave mean (SE) values and p-values. BMI = body mass index, DZ = dizygotic, concordant for PRS-enriched BMI = small within-twin-pair differences in body mass index (< 3 kg/m^2^) considering both observed and predicted BMI, PRS = polygenic risk score, n = number of pairs, ΔBMI = BMI_2011_ – BMI_1975_, ΔBMI P = p-values for BMI_2011_ – BMI_1975_ by co-twin group.

Table S14b: Adjusted values from contrast tests that compared observed body mass index at each timepoint within subgroups of dizygotic twin pairs concordant for PRS-enriched BMI

| **Observed BMI (kg/m^2^) in DZ twin pairs concordant for PRS-enriched BMI** | | | | | | | | | | | | |
| --- | --- | --- | --- | --- | --- | --- | --- | --- | --- | --- | --- | --- |
|  | DZ pair below prediction  (n = 162) | | | | DZ pair within prediction  (n = 702) | | | | DZ pair above prediction  (n = 113) | | | |
|  | Co-twin  with  lower BMI | Co-twin  with  higher BMI | Contrast | p-value | Co-twin with  lower BMI | Co-twin with  higher BMI | Contrast | p-value | Co-twin with  lower BMI | Co-twin  with  higher BMI | Contrast | p-value |
| 1975 | 19.9 (0.1) | 20.8 (0.1) | 0.9 (0.1) | <0.001 | 22.4 (0.1) | 23.4 (0.1) | 1.0 (0.04) | <0.001 | 27.0 (0.2) | 28.3 (0.2) | 1.2 (0.1) | <0.001 |
| 1981 | 20.7 (0.2) | 21.6 (0.2) | 0.9 (0.1) | <0.001 | 23.3 (0.1) | 24.0 (0.1) | 0.7 (0.1) | <0.001 | 27.9 (0.3) | 28.6 (0.3) | 0.7 (0.3) | 0.014 |
| 1990 | 21.9 (0.2) | 22.9 (0.2) | 1.0 (0.2) | <0.001 | 24.5 (0.1) | 25.2 (0.1) | 0.7 (0.2) | <0.001 | 28.9 (0.4) | 30.1 (0.4) | 1.2 (0.4) | 0.005 |
| 2011 | 24.3 (0.4) | 25.4 (0.4) | 1.1 (0.6) | 0.066 | 26.8 (0.2) | 27.5 (0.2) | 0.7 (0.3) | 0.012 | 32.0 (0.7) | 32.7 (0.7) | 0.7 (1.0) | 0.45 |
| ΔBMI | 4.4 [3.8, 5.0] | 4.5 [4.0, 5.1] |  |  | 4.4 [4.1, 4.7] | 4.1 [3.9, 4.4] |  |  | 4.9 [4.0, 5.9] | 4.4 [3.4, 5.4] |  |  |
| ΔBMI *P* | <0.001 | <0.001 |  |  | <0.001 | <0.001 |  |  | <0.001 | <0.001 |  |  |

BMI values are mean (Delta-method std. err.), ΔBMI values are mean [95% confidence interval], and contrast tests gave mean (SE) values and p-values. BMI = body mass index, DZ = dizygotic, concordant for PRS-enriched BMI = small within-twin-pair differences in body mass index (< 3 kg/m^2^) considering both observed and predicted BMI, PRS = polygenic risk score, n = number of pairs, ΔBMI = BMI_2011_ – BMI_197_, ΔBMI P = p-values for BMI_2011_ – BMI_1975_ by co-twin group.

Table S15a: Unadjusted values from post-hoc tests of partial interaction between co-twin subgroup and body mass index between adjacent timepoints in monozygotic and dizygotic twin pairs concordant for PRS-enriched BMI

|  | **BMI differences between co-twins and adjacent timepoints in twin pairs concordant for PRS-enriched BMI** | | | | | | | | | | | | |
| --- | --- | --- | --- | --- | --- | --- | --- | --- | --- | --- | --- | --- | --- |
|  | MZ pair below prediction  (n = 136) | | MZ pair within prediction  (n = 432) | | MZ pair above prediction  (n = 80) | | DZ pair below prediction  (n = 162) | | DZ pair within prediction  (n = 702) | | DZ pair above prediction  (n = 113) | |  |
|  | Contrast | p-value | Contrast | p-value | Contrast | p-value | Contrast | p-value | Contrast | p-value | Contrast | p-value |  |
| 1975 vs 1981, kg/m^2^ | -0.6 (0.4) | 0.091 | -0.2 (0.3) | 0.42 | -0.7 (0.8) | 0.39 | 0.03 (0.3) | 0.94 | -0.3 (0.2) | 0.18 | -0.6 (0.6) | 0.31 |  |
| 1981 vs 1990, kg/m^2^ | -0.2 (0.4) | 0.63 | -0.03 (0.3) | 0.92 | 0.2 (0.8) | 0.85 | -0.2 (0.4) | 0.62 | -0.001 (0.2) | 1.0 | 0.2 (0.6) | 0.79 |  |
| 1990 vs 2011, kg/m^2^ | 0.2 (0.5) | 0.71 | -0.4 (0.3) | 0.23 | 0.9 (1.1) | 0.40 | -0.02 (0.5) | 0.98 | -0.1 (0.3) | 0.68 | -0.4 (0.9) | 0.68 |  |

Values are mean (SE) and post-hoc tests of partial interaction gave p-values. BMI = body mass index, concordant for PRS-enriched BMI = small within-twin-pair differences in body mass index (< 3 kg/m^2^) considering both observed and predicted BMI, PRS = polygenic risk score, MZ = monozygotic, n = number of pairs, DZ = dizygotic.

Table S15b: Adjusted values from post-hoc tests of partial interaction between co-twin subgroup and body mass index between adjacent timepoints in monozygotic and dizygotic twin pairs concordant for PRS-enriched BMI

|  | **BMI differences between co-twins and adjacent timepoints in twin pairs concordant for PRS-enriched BMI** | | | | | | | | | | | |
| --- | --- | --- | --- | --- | --- | --- | --- | --- | --- | --- | --- | --- |
|  | MZ pair below prediction  (n = 136) | | MZ pair within prediction  (n = 432) | | MZ pair above prediction  (n = 80) | | DZ pair below prediction  (n = 162) | | DZ pair within prediction  (n = 702) | | DZ pair above prediction  (n = 113) | |
|  | Contrast | p-value | Contrast | p-value | Contrast | p-value | Contrast | p-value | Contrast | p-value | Contrast | p-value |
| 1975 vs 1981, kg/m^2^ | -0.7 (0.2) | <0.001 | -0.2 (0.1) | 0.037 | -0.8 (0.3) | 0.016 | 0.03 (0.1) | 0.80 | -0.3 (0.1) | 0.001 | -0.5 (0.3) | 0.060 |
| 1981 vs 1990, kg/m^2^ | -0.2 (0.2) | 0.22 | -0.02 (0.1) | 0.87 | 0.1 (0.4) | 0.76 | 0.1 (0.2) | 0.74 | -0.01 (0.1) | 0.94 | 0.5 (0.4) | 0.20 |
| 1990 vs 2011, kg/m^2^ | 0.9 (0.5) | 0.08 | -0.2 (0.3) | 0.41 | 0.8 (0.7) | 0.26 | 0.1 (0.5) | 0.89 | 0.01 (0.3) | 0.98 | -0.5 (1.0) | 0.61 |

Values are mean (SE) and post-hoc tests of partial interaction gave p-values. BMI = body mass index, concordant for PRS-enriched BMI = small within-twin-pair differences in body mass index (< 3 kg/m^2^) considering both observed and predicted BMI, PRS = polygenic risk score, MZ = monozygotic, n = number of pairs, DZ = dizygotic.
